# Atypical presentation of *Pneumocystis jirovecii* pneumonia in a patient with rheumatoid arthritis treated with tofacitinib: a case presentation

**DOI:** 10.1186/s41927-018-0042-7

**Published:** 2018-11-03

**Authors:** Ian F. J. Pirker, Jacqueline Krane-Nuber, Werner C. Albrich, Rüdiger Müller, Thomas Neumann

**Affiliations:** 1 Department of Internal Medicine, Cantonal Hospital St. Gallen, St. Gallen, Switzerland; 2 Division of Infectious Diseases & Hospital Epidemiology, Cantonal Hospital St. Gallen, St. Gallen, Switzerland; 3 Department of Rheumatology, Immunology and Rehabilitation, Cantonal Hospital St. Gallen, St. Gallen, Switzerland

**Keywords:** *Pneumocystis jirovecii* pneumonia, Rheumatoid arthritis, Tofacitinib, Hypercalcemia

## Abstract

**Background:**

We report the case of a patient with rheumatoid arthritis (RA) treated with tofacitinib who developed severe *Pneumocystis jirovecii* pneumonia (PJP) with an atypical clinical presentation.

**Case presentation:**

A 78-year old male patient with RA treated with tofacitinib, methotrexate (MTX) and low dose corticosteroids was admitted to the hospital with arthralgia and nausea. Laboratory findings revealed hypercalcemia with normal levels of parathyroid hormone (PTH) and elevated 1,25-(OH)_2_ vitamin D levels. A lung CT scan showed bilateral interstitial pneumonic infiltrates. PCR from bronchoalveloar lavage was positive for *Pneumocystis jirovecii*. Hypercalcemia resolved under PJP treatment and was – after exclusion of other possible causes – probably fungal associated.

**Conclusion:**

Due to the increased risk of opportunistic infections in immunocompromised patients, the finding of hypercalcemia in conjunction with a pulmonary infection should raise high clinical suspicion of PJP.

**Electronic supplementary material:**

The online version of this article (10.1186/s41927-018-0042-7) contains supplementary material, which is available to authorized users.

## Background

*Pneumocystis jirovecii* pneumonia (PJP) is a potentially life-threatening opportunistic fungal infection in immunocompromised patients. While it was an important opportunistic infection in the early HIV era prior to effective antiretroviral therapy, it is increasingly seen now in patients not infected with HIV with relevant morbidity and mortality [1]. However, non-HIV infected patients with PJP tend to have more acute presentations than HIV-infected patients [1].

Hypercalcemia can develop under several conditions, including hyperparathyroidism, osteolytic bone lesions or granulomatous diseases, but has also been described with infections, especially with PJP in patients with renal transplants [1].

We searched PubMed using following terms: *Pneumocystis jirovecii* AND hypercalcemia. Two case reports were listed. No literature on “hypercalc* AND rheumatoid arthritis AND *Pneumocystis (jirovecii* OR *carinii)* AND tofacitinib” or on “hypercalc* AND *Pneumocystis (jirovecii* OR *carinii)* AND tofacitinib” were identified.

Here we report the first case to our knowledge of a patient with RA treated with tofacitinib who presented with hypercalcemia as a leading symptom of PJP.

## Case presentation

A 78-year old male patient with RA treated with tofacitinib (10 mg p.o. daily), methotrexate (20 mg p.o. weekly) and low dose corticosteroids (prednisolone 5 mg p.o. daily), was admitted to hospital with a 2-week history of arthralgia, nausea and confusion (Additional file 1). RA was diagnosed 6 months ago, as the patient fulfilled the 2010 ACR/EULAR classification criteria with bilateral symmetric swollen and tender joints (wrists, hands and feet; over 10 affected joints in total) and arthralgia for 3 years (negative anti-CCP antibody and negative rheumatoid factor IgM). No signs of erosions were found on X-ray (hands and feet). Therapy was initiated with prednisone (20 mg/day) and MTX 15 mg/week s.c. with increasing doses over time. Tofacitinib was initiated 2 months prior hospital admission due to lack of efficacy of MTX monotherapy (MTX was switched to p.o. at that time, as the patient disliked injections).

At time of admission the patient reported shortness of breath on exertion but not at rest.

Physical examination showed following vital signs: temperature (auricular): 36.5 °C blood pressure: 178/95 mmHg, heart rate: 75 bpm, oxygen saturation: 88–90% at rest and on exercise 80% on room air. Wrists and ankles were swollen and tender bilaterally symmetric. There were normal findings on auscultation of heart and lungs without any other signs of venous congestion.

Laboratory findings on admission revealed hypercalcemia (albumin-corrected 3.12 mmol/l (normal range 2.0–2.6 mmol/l) and elevated 1,25- dihydroxyvitamin D levels (162 ng/l, normal range 22–111 ng/l). PTH was appropriately low at < 0.5 pmol/l (normal range < 1.3 pmol/l). Further laboratory findings are described in Table 1.

**Table 1:** parathyroid hormone, CRP: C-reactive protein

Laboratory parameter (units)	findings at admission	normal range
Calcium (mmol/L)	2.8	2.0–2.6
Albumine (g/l)	27.1	34–48
Intact PTH (ng/l)	15.3	15–65
PTH related peptide (pmol/l)	< 0.5	< 1.3
1,25-dihyroxyvitamin D (ng/l)	162	22–111
25-OH vitamin D total (mmol/l)	100	75–250
CRP (mg/l)	148	< 5
Procalcitonin quantitative (microg/l)	0.29	< 0.5

**Fig. 1:**
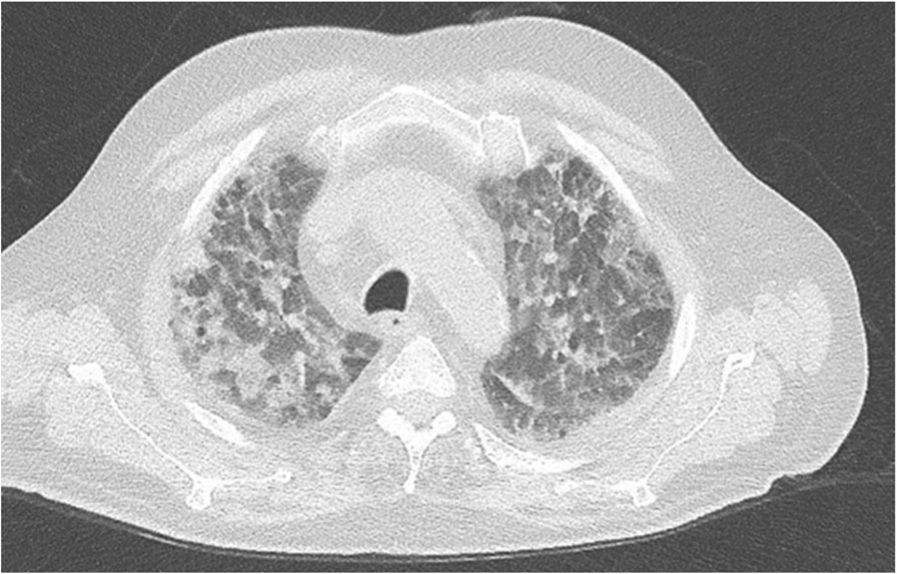
CT scan with bilateral pneumonic infiltrates

Further investigation of hypercalcemia included a whole body CT scan, which showed bilateral interstitial pneumonic infiltrates without pleural effusions (Fig. 1). The patient was referred from normal ward care to the ICU due to acute respiratory failure requiring non-invasive ventilation/ high-flow oxygen therapy. PCR from bronchoalveloar lavage (BAL) was positive for *P. jirovecii* and Rhinovirus A/B/C and a diagnosis of PJP was made with colonization of Rhinovirus. Adequate therapy with TMP/SMX i.v. (15 mg/kg/d) and prednisolone p.o. (1 mg/kg/d) was initiated. Treatment with TMP/SMX was stopped after 10 days because of severe hyponatremia (drop from 136 mmol/l to 119 mmol/l; normal range 136–144 mmol/l) and acutely worsening confusion, which was attributed most likely to acute hyponatremia or as a direct adverse drug reaction to TMP/SMX. As second line therapy we initiated clindamycin and primaquine. Confusion improved but thrombocytopenia developed (drop from 441 G/l to 83 G/l) 4 days later, which was felt to be due to primaquine and led to the discontinuation of clindamycin and primaquine.

Both the patient’s clinical condition and hypercalcemia were improved after 17 days of antibiotic therapy. PJP- prophylaxis with monthly inhaled pentamidin (300 mg) was initiated.

## Discussion and conclusion

We report for the first time a case of PJP in a tofacitinib treated RA patient that led to hypercalcemia. Physiologically, 1- alpha hydroxylation of 25-OH vitamin D occurs primarily in the proximal renal tubule in response to PTH [2]. This step should have been inhibited fully in our patient by the low serum PTH value and the high serum calcium and normal phosphate. Extrarenal 1-alpha hydroxylation of 25-OH vitamin D occurs in macrophages driven by interferon-gamma (IFN-γ) in neoplastic, granulomatous and infectious diseases [3]. This latter mechanism most likely explains the hypercalcemia associated with high 1,25-dihydroxyvitamin D levels.

It is postulated that in infectious diseases granuloma-forming activated macrophages can increase the conversion of 25-OH vitamin D to 1,25- dihydroxyvitamin D [3]. Normal macrophages do not produce 1,25-dihydroxyvitamin D, but 1-alpha hydroxylation of 25-OH vitamin D can be induced in macrophages exposed to IFN-γ, which is elevated in patients with PJP as shown in a study with pediatric patients [4].

In general, patients with RA are at increased risk to acquire infection in comparison to non RA study subjects due to the use of immunosuppressive therapy and/or the immunomodulatory effects of RA itself [5]. Long term safety reports regarding opportunistic infections (including PJP) among patients with RA using tofacitinib revealed a risk of infection, although the incidence rate is low and it is uncertain if this risk differs from that observed with bDMARDs [6, 7].

Recommendations on PJP prophylaxis in HIV negative patients with rheumatoid arthritis vary by author and region. A review by Wolfe et al. did not recommend PJP prophylaxis in patients with RA considering the low incidence of PJP in European and American patients [8].

In contrast, Mori et al. recommended short-term prophylaxis with TMP/SMX for patients with RA and newly treated with a biological and/or non-biological therapy [9].

A commonly encountered criterion to indicate PJP prophylaxis in non-HIV infected patients is receipt of prednisone ≥20 mg/kg/d for ≥1 month in addition to a second immunosuppressive or cytotoxic medication or a T-cell defect [10].

A recent study by Yukawa et al. proposes a scoring system based on usage of MTX, old age, number of immunosuppressive drugs, and dosage of prednisone, as these parameters were identified as risk factors of PJP in patients with rheumatoid arthritis [11]. A retrospective study showed that TMP/SMX prophylaxis significantly reduced PJP incidence in patients with rheumatic diseases (especially in patients receiving high dose steroids (over 60 mg/day) [12]. Based on this study Winthrop et al. identified following risk factors for PJP in patients treated with glucocorticoids: lymphopenia, initial glucocorticoid dose > 60 mg/day, current use of cyclophosphamide, anti TNF or rituximab use and presence of dermatomyositis, granulomatosis with polyangiitis and microscopic polyangiitis, while considering PJP prophylaxis at lower glucocorticoid doses (15 mg) when risk factor are present [13].

However, an international survey revealed that in 2010 of 3150 consecutive members of the American College of Rheumatology (ACR), 30% of surveyed rheumatologist reported that they never prescribed prophylaxis [14].

Hypercalcemia in patients with RA treated with immunosuppressive drugs in accordance with a pulmonary infection should raise high suspicion of PJP. Considering the potentially fatal courses of PJP, empirical treatment with TMP/SMX should be started in patients with sufficient clinical suspicion and should not be delayed until diagnostic procedures are performed or test results return. Evidence-based guidelines for PJP prophylaxis for the patients with autoimmune or connective tissue disease are urgently needed.

## Additional file

Additional file 1: Timetable of patient care. (DOCX 94 kb)

## Abbreviations

ACR: American College of Rheumatology
BAL: Bronchoalveloar lavage
CRP: C-reactive protein
HIV: Human immunodeficiency virus
MTX: Methotrexate
PCP: *Pneumocystis jirovecii* pneumonia
PCR: Polymerase chain reaction
PTH: Parathyroid hormone
RA: Rheumatoid arthritis
TMP/SMX: Trimethoprim/sulfamethoxazole
