# Supplementary material for: Atypical presentation of Pneumocystis jirovecii pneumonia in a patient with rheumatoid arthritis treated with tofacitinib: a case presentation
Source: BMC Rheumatol. 2018 Nov 3;2:34. doi: 10.1186/s41927-018-0042-7 (PMC6390573; doi:10.1186/s41927-018-0042-7)
Supplement: Supplementary file 1 — Timetable of patient care. (DOCX 94 kb) [file 41927_2018_42_MOESM1_ESM.docx]

Treatment with clindamycin and primaqin and discontinuation of TMP/SMX due to adverse drug reaction

**Diagnosis of *pneumocystis jirovecii* pneumonia (PCP)**

Laboratory findings: hypercalcemia

Improvement of patient`s clinical condition and hypercalcemia

PCP prophylaxis with inhaled pentamidin

Day 1833ay 3

Discontinuation of clindamycin and primaqin due to adverse drug reaction

Day 1733ay 3

Day 1433ay 3

Day 433ay 3

Day 333ay 3

Day 1

Referral to intensive care unit

Inital treatment with TMP/SMX

78-year old male patient with a 6 month history of rheumatoid arthritis treated with tofacitinib (10mg p.o. daily), methotrexate (20mg p.o. weekly) and low dose corticosteroids (prednisolone 5mg p.o. daily)

Symptoms: arthralgia, nausea and confusion

CT Image: bilateral interstitial pneumonic infiltrates

Bronchoalveloar lavage (BAL) was positive for *P. jirovecii*

Oxygen saturation on room air: 88%-90%, no signs of venous congestion
